# Development of In Situ Methods for Preparing La-Mn-Co-Based Compounds over Carbon Xerogel for Oxygen Reduction Reaction in an Alkaline Medium

**DOI:** 10.3390/nano14161362

**Published:** 2024-08-19

**Authors:** Jhony Xavier Flores-Lasluisa, Bryan Carré, Joachim Caucheteux, Philippe Compère, Alexandre F. Léonard, Nathalie Job

**Affiliations:** 1 Department of Chemical Engineering—NCE (Nanomaterials, Catalysis, Electrochemistry), University of Liège, B6a, Allée du Six Août 13, 4000 Liège, Belgium; 2 Center for Applied Research and Education in Microscopy (CAREM), Chemistry Institute, University of Liège, B6c, Allée du Six Août 11, 4000 Liège, Belgium; 3 Interfaculty Research Center on Biomaterials (CEIB), Chemistry Institute, University of Liège, B6c, Allée du Six Août 11, 4000 Liège, Belgium; 4 Department of Chemical Engineering—CARPOR, University of Liège, B6a, Allée du Six Août 13, 4000 Liège, Belgium

**Keywords:** oxygen reduction reaction, in situ method, C-O-M covalent bonds, Co/MnO heterointerfaces, Co-N_x_-C species

## Abstract

Metal oxides containing La, Mn, and Co cations can catalyze oxygen reduction reactions (ORRs) in electrochemical processes. However, these materials require carbon support and optimal interactions between both compounds to be active. In this work, two approaches to prepare composites of La-Mn-Co-based compounds over carbon xerogel were developed. Using sol-gel methods, either the metal-based material was deposited on the existing carbon xerogel or vice versa. The metal oxide selected was the LaMn_0.7_Co_0.3_O_3_ perovskite, which has good catalytic behavior and selectivity towards direct ORRs. All the as-prepared composites were tested for ORRs in alkaline liquid electrolytes and characterized by diverse physicochemical techniques such as XRD, XPS, SEM, or N_2_ adsorption. Although the perovskite structure either decomposed or failed to form using those in situ methods, the materials exhibited great catalytic activity, which can be ascribed to the strengthening of the interactions between oxides and the carbon support via C-O-M covalent bonds and to the formation of new active sites such as the MnO/Co heterointerfaces. Moreover, Co-N_x_-C species are formed during the synthesis of the metal compounds over the carbon xerogel. These species possess a strong catalytic activity towards ORR. Therefore, the composites formed by synthesizing metal compounds over the carbon xerogel exhibit the best performance in the ORR, which can be ascribed to the presence of the MnO/Co heterointerfaces and Co-N_x_-C species and the strong interactions between both compounds. Moreover, the small nanoparticle size leads to a higher number of active sites available for the reaction.

## 1. Introduction

Fuel cell electric vehicles (FCEVs) have gained huge interest as future alternatives to conventional vehicles because of their higher efficiency and lower time of fueling [1]. Moreover, the use of hydrogen as fuel motivates the development of FCEVs because hydrogen can be produced from renewable energy sources, has a high gravimetric energy density, and produces no harmful emissions [2]. However, the electrochemical reactions that occur in these devices, such as the hydrogen oxidation reaction (HOR) and oxygen reduction reaction (ORR), exhibit sluggish kinetics, making efficient catalysts necessary. Among both reactions, the ORR has the worst kinetics and requires a higher catalyst loading. Currently, the most commonly used fuel cell systems are proton-exchange membrane fuel cells (PEMFCs), which operate under acidic conditions and use a polymer membrane as an electrolyte [1]. These electrochemical devices employ Pt-based materials for the ORR because of their great catalytic activity, but Pt is scarce and costly [3,4,5]. Therefore, preparing alternative materials based on abundant and cheap elements is compulsory for developing this technology. In this sense, working under alkaline conditions can increase the number of materials stable and active in ORR [6]. Moreover, the alkaline medium is also less corrosive, and the ORR kinetics are faster than in the acidic medium [7]. Hence, developing electrocatalysts for alkaline anion-exchange membrane fuel cell (AAEMFC) systems as an alternative to PEMFCs is an interesting challenge.

Perovskite-based materials with ABX_3_ structure [8] and Ruddlesden–Popper perovskites [9], which are layered derivatives of the ABX_3_ family, have been observed as promising alternative electrocatalysts because of their excellent physicochemical and electrochemical properties. In this sense, the perovskite metal oxides with formula LaBO_3_ (where B is a *3d* transition metal) have been reported to show great activity for ORR [10]. The catalytic activity depends on the *3d* transition metal that leads to different interactions between the catalytic B^3+^ species and the intermediate species involved in ORR [11,12]. Sunarso et al. [13] observed that the coexistence of two metals via doping can enhance the ORR performance, suggesting that the presence of both cations is beneficial for the catalytic activity. Among the different possible compositions, the LaMn_1−x_Co_x_O_3_ perovskite materials [14,15,16] were reported to show great activity towards ORRs; this high activity has been ascribed to the crystal structure, oxidation states of the *3d* transition metals, oxygen vacancies, and synergistic effect between both cations. During cation substitution, the concentrations of Mn^4+^ species and oxygen vacancies increase. This positively affects the ORR kinetics by enhancing the electron transfer and facilitating a moderate interaction between the surface active sites and intermediates [15,16]. However, recent studies showed [17] that it is not necessary to combine these cations within a unique structure to obtain good ORR performance. Indeed, it was observed that Mn-Co-based (hydr)oxides with low crystallinity stabilized with La^3+^ cation can provide a large number of active sites from lattice defects.

Despite the great advantages of the metal oxides in ORR, these materials display two main drawbacks that limit their performance: (i) low surface area and (ii) low electrical conductivity [18,19,20]. To solve these problems, the metal oxides are generally synthesized in a nanoscale shape to increase the active surface available for the reaction [21]; then, these nanoparticles are either supported or mixed with carbon materials aiming to increase the electrical conductivity, thereby favoring the electron transfer of the redox processes over the metal oxides [19,22,23]. Moreover, carbon materials can also participate in the ORR by acting as co-catalysts, providing hydrogen peroxide species for their further reduction and enhancing the overall reactions [24,25]. A simple method to prepare composites is through physical mixing using mortar or ball milling [14,26]. These methods can generate interactions between both materials via the C-O-B bond species that enhance the electron transfer. These interactions can be further strengthened to covalent bonds by mixing the materials via either high-temperature thermal [20] or low-temperature hydrothermal treatments [27].

A proper way to ensure the presence of strong interactions would be to prepare the perovskite metal oxide in the presence of carbon materials. In the literature, one can find examples of LaBO_3_ prepared over carbon materials, i.e., without the partial substitution of cation B^n+^ with another *3d* transition metal [28,29]. In this sense, Shi et al. [30] reported that the partial substitution of cation B^n+^ can lead to the collapse of the perovskite structure, generating poor catalytic crystal phases. However, other highly active structures or heterostructures can also be formed under an inert atmosphere. The thermal decomposition of La precursors can lead to the formation of La_2_O_3_ species [31] that can form C-O-La covalent bonds with the carbon material [32]. These bonds greatly influence the adsorption of oxygen and the desorption of the surface-adsorbed hydroxide, which is a rate-determining step for ORRs [32]. On the contrary, Co precursors can decompose to form metallic Co nanoparticles, which, in the presence of N-doped carbon materials, can result in the Co-N active sites [33,34,35,36]. Qian et al. [33] concluded that the active sites are Co-N species and not the metallic Co or Co_3_C species that could negatively affect the activity. Tang et al. [34] suggested that pyridinic N can coordinate with Co atoms to form Co-N_x_-C species, which optimizes the chemisorption of intermediates and facilitates electron transfer. Meanwhile, Amiinu et al. [35] demonstrated that pyridinic N could activate the neighboring C atoms, while the Co atoms can adsorb O_2_ and transfer electrons to N, lowering the energy barrier. Alternatively, Mn precursors can lead to the formation of MnO/C composites that exhibit high activity in ORRs [37,38]. Shi et al. [37] claimed that the activity resulted from the synergistic effect between both materials that enhances the charge transfer. In another study, however, Mishra et al. [38] indicated that good activity is ascribed to the optimum metal oxide loading, which avoids the agglomeration of the active sites. Interestingly, MnO can interact with Co-N_x_ species to generate Mn-Co-N_x_ catalytic species that accelerate the ORR by weakening the adsorption of adsorbed species [39]. Regarding these catalytic interactions, several studies have reported the remarkable ORR performance of Co/MnO heterointerfaces [40,41,42,43,44]. In that regard, Wang et al. [40] attributed the good ORR activity of the Co/MnO heterointerfaces to improved electrical conductivity and promoted adsorption of the oxygenated intermediates provided by metallic Co and MnO, respectively. Niu et al. [41] reported that the heterointerfaces between these two components optimize their electronic structure and enhance their electronic conductivity. This fact reduces the energy barrier for adsorption/desorption of the intermediates of the reaction, accelerating the ORR. Meanwhile, Guo et al. [42] described that these heterointerfaces promote charge transfer and also provide extra electrons for the ORR reaction. These heterointerfaces are directly related to grain boundaries, which are considered potential sites for electrochemical reactions because these can lead to additional catalytic enhancements [45]. Moreover, it cannot discard a synergistic effect between the existing phases, which considerably promotes the electrochemical reaction [46].

Another alternative method to ensure the presence of perovskite structure could be the synthesis of the carbon material onto an existing perovskite. This approach was already accomplished by preparing carbon nanotubes over a LaNiO_3_ structure using the chemical vapor deposition (CVD) method [47]. However, the conditions involved in the preparation of carbon materials could also affect the stability of the perovskite structure, leading to the formation of the above-mentioned crystal structures.

Among carbon materials, carbon xerogels demonstrate exceptional features in terms of pore structure and electrical conductivity, making them ideal supports for electrocatalysis [48]. Previous studies [49,50] obtained good electrocatalytic materials in ORR. This performance was ascribed to the porosity of carbon xerogels and the interaction between the active sites and the carbon material. Concerning the pore texture, micropores can increase the intrinsic activity because these strongly adsorb oxygen, thus weakening the O-O bond producing the ORR [51]. Meanwhile, mesopores are important in providing surface area for the deposition of the active phase and favoring mass transfer processes [50]. In this sense, it is feasible to tailor the pore texture of the carbon xerogel by tuning the composition of the precursor solution [52]. The ideal pore size suitable for fuel cell applications should be between 50 and 100 nm. This leads to the formation of large mesopores, which minimize mass transfer limitations [53].

In this work, we propose two in situ synthesis methods for preparing composites of LaMn_0.7_Co_0.3_O_3_ perovskite (P) and carbon xerogel (CX) with great activity towards oxygen reduction reactions (ORRs). In the first approach, the metal oxide was synthesized via a sol-gel method in the presence of CX powder (particle size around 10 µm). Meanwhile, in the second one, CX was synthesized by the polycondensation of resorcinol and formaldehyde in the presence of P. Moreover, a composite was prepared for comparison by physically mixing both P and CX materials using an agate mortar. The different as-prepared composites and the pristine materials were characterized by microscopic, spectroscopic, and diffraction techniques. To determine the activity in the ORR, polarization curves were measured using a rotating ring–disk electrode. The goal of this work is to demonstrate that in situ methods can generate stronger interactions between components, resulting in highly active sites that significantly enhance the ORR. Finally, the characterization of the pristine materials and the mixed composite will also provide information about the importance of mixing carbon material and metal oxide to enhance the ORR significantly.

## 2. Experimental

### 2.1. Materials and Reagents

The reagents used in this work were lanthanum (III) nitrate hexahydrate (La(NO_3_)_3_O_3_·6H_2_O—Sigma Aldrich, St. Louis, MO, USA, 99 wt.%), manganese (II) nitrate hydrate (Mn(NO_3_)_2_·xH_2_O—Sigma Aldrich, 98 wt.%), cobalt (II) nitrate hexahydrate (Co(NO_3_)_2_·6H_2_O—Sigma Aldrich, 98 wt.%), ethylenediaminetetraacetic acid (EDTA—Sigma Aldrich, ACS reagent), citric acid (Sigma Aldrich, 99 wt.%), resorcinol (Merck, Rahway, NJ, USA, 35 wt.%), formaldehyde stabilized by 10–15 wt.% methanol (Sigma Aldrich, ACS reagent, 37 wt.%), sodium carbonate (Na_2_CO_3—_Sigma Aldrich, ACS reagent, 99,5 wt.%), ammonia (NH_3_—VWR Chemicals, Radnor, PA, USA, analytic reagent), hydrochloric acid (HCl—Sigma Aldrich, ACS reagent, 37 *v*/*v*), potassium nitrate (KNO_3—_Sigma Aldrich, ACS reagent), and potassium hexacyanoferrate (III) (K_3_[(Fe(CN)_6_]—Merck, ACS reagent).

Moreover, 2-propanol 100% (VWR), Nafion^®^ 5 wt.% solution (Quintech, Göppingen, Germany), potassium hydroxide (KOH—Supelco, St. Louis, MO, USA, 85 wt.%), and 20 wt.% Pt/C (Sigma-Aldrich) were also used. The 20 wt.% Pt/C commercial material was used as a reference catalyst towards the ORR. All solutions were prepared with ultrapure water (18 MΩ cm^−1^ from a Veolia water system). The gases N_2_ (99.999%) and O_2_ (99.995%) were provided by Air Liquide (Paris, France). All the reagents used in this work were used without any additional treatment.

### 2.2. Synthesis Procedure

The LaMn_0.7_Co_0.3_O_3_ perovskite was synthesized by a modified sol-gel method described elsewhere [54]. The molar ratio employed for this synthesis was 2:3:1:1 for EDTA, citric acid, lanthanum precursor, and the combined manganese and cobalt precursors, respectively. As the synthesis was prepared for 3 g of the final product, 0.25 mol of EDTA was dissolved in a mixture of 123 mL ultrapure water and 9.9 mL NH_3_. Then, the corresponding amount of the metal precursors and citric acid were added to the EDTA solution. The stable sol complex was formed by slowly adding NH_3_ to adjust the pH to 9. After stirring the solution at 80 °C for 6 h, it was dried in the oven overnight at 150 °C. Next, the material was ball-milled employing a Fritsch planetary mill (Mono Mill P6) in an agate jar with 1 cm diameter agate balls with a ratio of 3 g of material per 20 balls. The procedure consisted of 24 cycles of grinding at 400 rpm for 1 min each, followed by 15 s of pause [55]. Finally, the product was calcined at 500 °C for 1 h and at 700 °C for 6 h to form the perovskite metal oxide material, which is named P hereafter.

The carbon xerogel was synthesized from polycondensation of resorcinol and formaldehyde in water following the method described by Job et al. [56]. First, formaldehyde (F) was added to a solution of water and resorcinol (R), the *R/F* molar ratio being equal to 1:2. The mixture was stirred for 1 min at room temperature. Then, sodium carbonate (C) was added to the solution to reach a *R/C* molar ratio equal to 450. The pH of the solution was 5.4. The molar dilution ratio *D* (water/reactants) used for the synthesis of the carbon xerogel was chosen equal to 5.7. It is important to note that the water contained in the formaldehyde solution is also included for the determination of the ratio *D*. Then, the resulting solution was sealed in an autoclavable glass flask and kept at 85 °C for 72 h for the gelation and aging. To dry the xerogel, it was subjected to vacuum evaporation (9.2 kPa) for 32 h at 60 °C. Then, the pressure was decreased progressively to 2 kPa and kept for 46 h. Previous studies [57] reported that grinding the dry polymer gel before pyrolysis does not affect the specific surface area and prevents the formation of fine particles. Thus, the dry polymer gel was ball-milled using the same protocol described above to reach a particle size of around 10 µm. The thermal treatment was performed in a tubular oven under nitrogen flow at 800 °C for 2 h following a previous procedure [57]. The as-prepared carbon xerogel was named CX.

The composites containing metal oxides and carbon xerogel were synthesized via in situ methods using two different approaches. In both routes, the objective was to reach ~20 wt.% of the metal-based compound to have an active phase concentration similar to the commercial catalyst.

The first approach consisted of synthesizing the metal oxide in the presence of CX particles using the sol-gel method described above with some modifications. The concentration of the reactants was adjusted to obtain 0.2 g of the final metal oxide product. However, the water amount did not decrease; it increased up to 40 mL to favor mixing with CX. Then, 0.8 g of CX powder was added to the solution after the pH was adjusted to 9. Then, the suspension was also stirred at 80 °C for 6 h to evaporate the solvent. The resulting material was dried at 150 °C in the oven overnight. Finally, the sample was thermally treated in a tubular oven under a nitrogen atmosphere: the temperature was increased up to 700 °C at a heating rate of 5 ºC min^−1^, and the final temperature was kept for 6 h. The as-prepared sample was named CX_P_N_2_. To prepare the sample CX_P_O_2_, the CX_P_N_2_ sample was further treated under air at 250 °C for 4 h.

The second approach consisted of synthesizing CX in the presence of perovskite using the same method described for the CX synthesis. However, instead of adding sodium carbonate, the required amount of perovskite to reach 20 wt.% in the final hybrid material was added. The pH of the solution was measured and equaled 5.6 at that point. Then, the aging, drying, and pyrolysis steps were performed similarly as in the CX synthesis procedure. The sample obtained was named P_CX_5.6. To favor the formation of adequate porosity, another sample was synthesized following the above procedure, but, in this case, the pH was decreased to 5.3 by a dropwise addition of 0.5 M HCl aqueous solution. Indeed, it is known that the pH of the precursor solution of CX influences the final pore sizes [52], which is the reason why the pH was decreased, such as to be close to the value of 5.4 measured for the CX prepared above. This process resulted in the sample P_CX_5.3.

In addition, a reference composite was prepared by physically mixing the metal oxide with carbon xerogel in an equal mass ratio; the mixing was performed by hand milling in an agate mortar. This mass ratio was reported to provide the best ORR performance with the LaMn_0.7_Co_0.3_O_3_ perovskite [14]. The sample is named P + CX hereafter.

### 2.3. Characterization Techniques

The different materials were characterized by X-ray diffraction (XRD) using a Bruker D8 Twin-Twin diffractometer (Billerica, MA, USA) with a Cu K_α_ radiation source in the 2θ range from 10° to 80° with a step of 0.02°. The data analysis was performed with the Diffract. Eva software 5.3 uses references from the PDF4+ database (International Center for Diffraction Data). The morphology of the as-prepared samples was characterized by scanning electron microscopy (SEM) in a FEG-SEM Tescan Clara (TESCAN, Brno, Czech Republic) at 15 kV of accelerating voltage under high vacuum. The samples were gold-coated in a sputtering device (Balzers, SCD004 sputter coater, Vaduz, Liechtenstein). The microstructure of the samples was characterized by transmission electron microscopy (TEM). The samples were deposited on 200 mesh carbon-coated Cu-grids (S160, AGAR) and observed in a TEM/STEM TECNAI G2 Twin microscope (FEI, Eindhoven, The Netherlands) operating at 200 kV.

The samples were characterized by X-ray photoelectron spectroscopy (XPS) in a VG-Mictrotech Multilab 3000 equipment (Thermo-Scientific, Waltham, MA, USA) equipped with an Al K_α_ radiation source (1253.6 eV). The XPSPEAK41 software (https://xpspeak.software.informer.com/4.1/, accessed on 16 August 2024) was used to deconvolve XPS data. A combination of Lorentz–Gaussian functions was used to fit these data along with a Shirley line for background correction.

The textural properties of the samples were determined by physical N_2_ adsorption–desorption measurements at −196 °C using an ASAP 2420 device (Micromeritics, Norcross, GA, USA) equipped with a high-vacuum turbomolecular pumping system. Before analysis, the samples were degassed under a high vacuum (2 × 10^−4^ Pa) for 5 h at 30 °C followed by 2 h at 270 °C. The specific surface area (*S*_BET_) and total micropore volume (*V*_DR_,_N2_) were determined by applying the Brunauer–Emmett–Teller (BET) method and the Dubinin–Radushkevich (DR) equation to the N_2_ adsorption isotherm, respectively [58]. The mesopore volume (*V*_meso_) was calculated by subtracting the micropore volume from the volume of N_2_ adsorbed at a relative pressure of 0.95.

The carbon content in the composite materials was determined by thermogravimetric (TG) measurements using Sensys Evo TG-DSC equipment (Setaram, Caluire, France). The samples were heated to 800 °C at a heating rate of 5 °C min^−1^ under an N_2_ flow of 20 mL min^−1^ and kept at that temperature for a further 2 h to reach a constant mass.

### 2.4. Electrochemical Characterization

First, an ink was prepared by sonicating a suspension with 19.9 mg of the sample with 5.15 mL of a solution (70 vol% water, 27 vol% isopropanol, and 3 vol% Nafion^®^). The electrochemical measurements were performed in a three-electrode cell in 0.1 M KOH solution at 25 °C; the temperature was controlled using a double sheath cell and a thermostatic bath. The bipotentiostat employed for the measurements was an Autolab PGSTAT30 (Metrohm, Barendrecht, The Netherlands). The working electrode was a rotating ring–disk electrode (RRDE) from Metrohm consisting of a glassy carbon (GC) disk (5 mm diameter) and a Pt ring. A platinum sheet electrode was used as a counter-electrode and an Ag/AgCl electrode (3 M KCl) as a reference electrode; Metrohm provided both electrodes. All the potentials measured were converted to standard relative hydrogen reversible (RHE) potentials using the Nernst equation:

(1) $$E_{RHE}=E_{Ag/AgCl}+0.209+0.591pH$$

Cyclic voltammetry (CV) and linear sweep voltammetry (LSV) measurements were performed for electrochemical characterization of the materials. A volume of 115 µL of the prepared ink was deposited onto the GC disk electrode to form a uniform catalytic layer of 590 µg cm^−2^ active material. CVs were performed after 20 min of bubbling either N_2_ or O_2_ into the KOH solution. The voltammograms were obtained from 0 to 1 V (vs. RHE) at 10 mV s^−1^.

Polarization curves were performed to study the ORR at 1600 rpm at 2 mV s^−1^ from 1 to 0 V (vs. RHE) in an O_2_-saturated 0.1 KOH solution. The Pt ring was kept at 1.5 V (*vs.* RHE) throughout the measurements. The number of electrons transferred ($n_{e^{-}}$) during the reaction was calculated from the oxidation of the hydrogen peroxide at the Pt ring electrode using the following equation [59]:

(2) $${HO}_{2}^{-}\left[\%\right]=200\times \frac{I_{ring}/N}{I_{disk}+I_{ring}/N}$$

(3) $$n_{e^{-}}=\frac{4I_{disk}}{I_{disk}+I_{ring}/N}$$

where I_disk_ and I_ring_ are the currents measured at the disk and ring, respectively, and N is the collection efficiency of the ring that was experimentally determined to be 0.265. To obtain this value, it was necessary to prepare a solution of (K_3_[Fe(CN)_6_)]) 0.1 M in (KNO_3_)1 M. Then, polarization curves were performed at different rotation speeds (400–2025 rpm) at 5 mV s^−1^ from 1.4 to 0 V (vs. RHE) in an N_2_-saturated solution [60]. When the potential in the disk electrode is decreased, a limiting current is achieved from the reduction process of Fe^3+^ to Fe^2+^. At the same time, the high potential of the ring produces the reverse process, i.e., the oxidation process of Fe^2+^ to Fe^3+^ also generates a limiting current. The N parameter is determined as a relation between the limiting current of both electrodes according to the following equation [60]:

(4) $$N=\frac{-I_{ring}}{I_{disk}}$$

The N value is obtained from an average of all the values calculated at different rotation speeds.

All the current densities measured are further presented per the geometric surface of the glassy carbon electrode.

## 3. Results and Discussion

### 3.1. Structural Characterization

**Figure 1:**
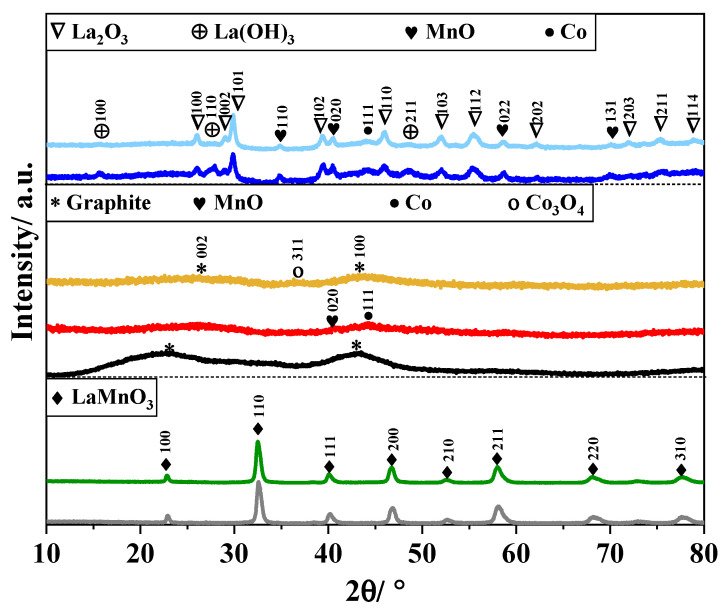
X-ray diffraction patterns for the pristine perovskite (P), carbon xerogel (CX), and composite materials. (**▬**) P; (**▬**) P + CX; (**▬**) CX; (**▬**) CX_P_N_2_; (**▬**) CX_P_O_2_; (**▬**) P_CX_5.6; (**▬**) P_CX_5.3.

Since the crystal structure can strongly influence the electrocatalytic activity of metal oxides, an appropriate characterization of the composites was performed using the X-ray diffraction technique, and the XRD patterns are displayed in Figure 1. The samples P and P + CX show similar diffraction peaks at 2θ values close to 23°, 33°, 40°, 47°, 58°, and 68° that can be indexed to a cubic lanthanum manganite perovskite phase (LaMnO_3_ (Pm3m), PDF code: 00-051-1516) [61]. As expected, the physical mixing to form the P + CX composite does not cause any structural change in the perovskite structure.

The diffractogram of the CX material features two broad peaks at 2θ equal to 25° and 43° that can be indexed to graphite (C, PDF code: 00-056-0159) [62]. Such broad peaks are characteristic of disordered carbons with very small graphitic domains. These diffraction peaks are also observed in the CX_P-based materials, indicating the presence of carbon material. Apart from the CX diffraction peaks, it is hard to identify other crystal structures because of the weak signals, which are ascribed to the amorphous phase or low crystallinity of the metal-based materials. However, two small peaks at around 41° and 44° can be distinguished in the CX_P_N_2_ sample; those peaks could be indexed to cubic manganese (II) oxide (MnO (Fm3m), PDF code: 04-005-4310) [63] and cubic metallic cobalt (Co (Fm3m), PDF code: 00-015-0806) [64] phases, respectively. The metallic Co compound might be formed from the reduction of Co^2+^ species with the reducing gases generated during the decomposition of the chelating agents (e.g., H_2_ and CH_4_) [65]. The perovskite structure is not formed, but the Co/MnO heterointerfaces could be formed from the interaction between MnO and Co, which are highly electrocatalytic for ORR [40,66]. These heterointerfaces especially enhance electrochemical reactions by promoting electron transfer and optimizing the chemisorption energies for the reaction intermediates involved in the ORR [41]. Figure S1a shows in more detail the CX_P_O_2_ sample, which displays important changes in the crystal structures. Small peaks at 36°, 60°, and 65° can be observed, indicating the formation of cubic cobalt (II, III) oxide (Co_3_O_4_ (Fd-3m), PDF code: 00-042-1467) [64] to the detriment of the Co phase. The Co_3_O_4_ crystal structure was also reported to be catalytically active [67]. Although no peak corresponding to Mn-based structures can be discerned, partial oxidation of MnO cannot be discarded.

Concerning the P_CX-based composites, the XRD patterns clearly show the decomposition of the perovskite structure during the preparation of CX; this can be ascribed to low pH during mixing, autoclave conditions during gelling and high temperature during pyrolysis. Apart from the MnO and Co, one can identify lanthanum (III) oxide (La_2_O_3_) and lanthanum (III) hydroxide (La(OH)_3_) crystal structures. The peaks at 2θ values close to 26°, 30°, 39°, 46°, 52°, and 55° can be indexed to hexagonal La_2_O_3_ ((P-3m1), PDF code: 04-005-4229) [68], while the peaks at 16°, 28°, and 49° are related to hexagonal La(OH)_3_ ((P6_3_/m), PDF code: 04-005-8587) [69]. Figure S1b shows that the intensity of La(OH)_3_ peaks decreases when the synthesis pH decreases slightly; thus, a higher concentration of the La_2_O_3_ phase is expected in this composite. This fact confirms that the addition of HCl reduces the concentration of H_2_O molecules, which react with the La_2_O_3_ phase to form the La(OH)_3_ phase [70].

The crystallite size of the different crystal phases was also determined by the Scherrer equation [71], and the results are summarized in Table S1. The perovskite structure displays a crystallite size of around 25 nm, but this value decreases for the CX_P-based materials; this probably results from the decomposition of the pristine structure to form other crystal phases. The CX_P-based composites display crystallite sizes of around 20 nm for La_2_O_3_ and MnO, whereas one obtains ~10 nm for La(OH)_3_ and Co. For the P_CX-based composites, it was not possible to determine any crystallite size because of the low intensity of the diffraction peaks.

**Figure 2:**
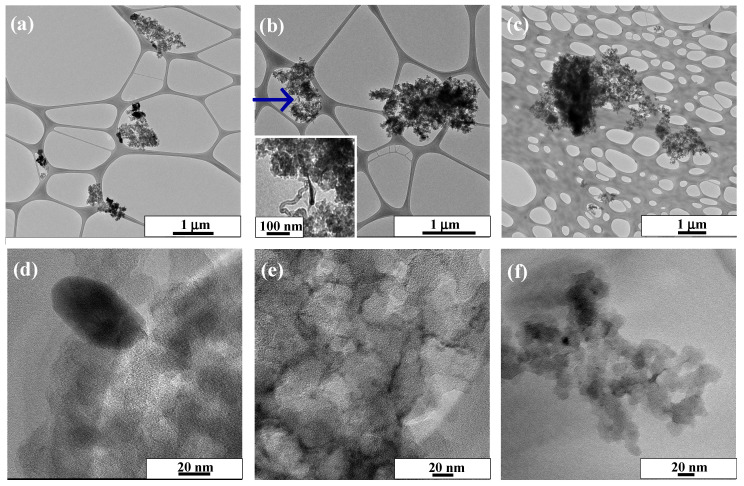
TEM images for the different samples: (**a**,**d**) P + CX; (**b**,**e**) CX_P_N_2_; and (**c**,**f**) P_CX_5.3. Inset (**b**): magnification of the region indicated by the arrow.

To obtain more information about the microstructure and the distribution of the metal-based compounds over the carbon xerogel, the P + CX, CX_P_N_2_ and P_CX_5.3 samples were characterized by TEM. Figure 2 shows the TEM images of the different samples at different scales. In the P + CX sample, it is easy to distinguish the perovskite and CX components (Figure 2a,d). The nanoparticles have particle sizes ranging from 24 to 76 nm, and form agglomerates that come into contact with the carbon material, either completely or partially. This contact is important for improving the electrical conductivity of the composite. In the case of the composites prepared via in situ methods, one cannot distinguish clearly both components, which suggests a good dispersion of the metal-based components. Interestingly, the CX_P_N_2_ sample (Figure 2b) develops carbon nanotubes (blue arrow) when the chelating agents and cobalt-containing precursors are thermally treated under an inert atmosphere. In a previous study, it was indicated that the decomposition of chelating agents leads to carbon-containing species that react over the cobalt sites, hence producing carbon nanotubes [72]. Unfortunately, XRD does not detect these carbon structures because of their low concentration. The metal-based nanoparticles seem well-distributed over CX, with a nanoparticle size ranging between 4 and 8 nm. For the P_CX_5.3 sample, the microstructure is similar to the sample synthesized via the previous route, i.e., the sample is homogenous, and the nanoparticles are well-distributed. However, the big difference is the nanoparticle size, which increases to the 10–20 nm range. The preparation of metal-based materials over CX favors the formation of well-distributed small nanoparticles, potentially enhancing the electrocatalytic performance.

### 3.2. Surface Morphology and Textural Characterization

**Figure 3:**
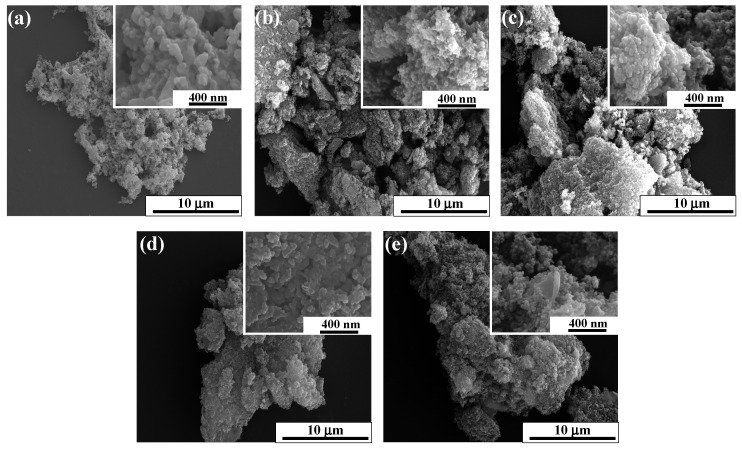
SEM images for the different samples: (**a**) pristine perovskite (P); (**b**) CX; (**c**) P + CX; (**d**) CX_P_N_2_; and (**e**) P_CX_5.3.

Generally, porous structures are desired for electrocatalytic applications because this can help the diffusion of the electrolyte through the material, enabling its interaction with all the active sites. This also favors the access of the reactants to the active site and the release of the products. Thus, to characterize the surface morphology, some materials were examined by SEM, and the results are displayed in Figure 3. The pristine perovskite shows compacted aggregates (~1 µm) with voids that probably result from the thermal decomposition of the chelating agents during the calcination treatment (Figure 3a) [54]. On the contrary, the CX-containing materials show a sponge-like shape consisting of agglomerated compacted aggregates. Despite their similar morphology, slight differences can be distinguished related to the size of existing aggregates of particles and their compactness. For the CX sample (Figure 3b), it is noticed that the aggregates are heterogeneous, with different sizes, from 1 to 8 µm. Quite similar surface morphology is observed for the P + CX composite (Figure 3c), indicating that no important changes are observed after the physical mixing. In the case of the composites prepared by in situ methods, both composites are mainly made of large aggregates (~20 µm). Regarding the shape, the P_CX_5.3 sample (Figure 3e) displays the same spongy morphology as the other composites. On the contrary, the CX_P_N_2_ sample (Figure 3d) has a more irregular and rough shape. This might result from the deposition of carbon material over the CX nodules. This carbon material comes from the thermal decomposition of the chelating agents under an inert atmosphere. As an alternative explanation, one cannot discard the partial surface erosion of the CX nodules caused by the gases released during the decomposition of metal precursors and chelating agents while forming the metal oxides [72]. The insets of the main images exhibit how the particle size of the composites can change depending on the synthesis procedure. First, the perovskite is made of particles with an irregular shape ranging roughly between 50 and 200 nm; those particles are agglomerated. Meanwhile, the CX sample is composed of connected micropore nodules of around 30–50 nm size with voids in-between, which contribute to the meso-macroporosity. Therefore, the final pore texture of the carbon xerogels depends on the sum of the micropore and meso-macroporosity volumes created on the nodules and the interspaces between the carbon nodules, respectively [73]. In the P + CX sample (inset in Figure 3c), particles of different sizes can be distinguished. These particles are related to carbon nodules and perovskite particles. Thus, this suggests a good dispersion of the metal oxide over the carbon material. As was already mentioned, the formation of the metal compounds in the carbon xerogel to form the CX_P_N_2_ sample strongly affects its morphology. It can be observed that the size of carbon nodules increases slightly (~40–100 nm, see inset in Figure 3d), which might be related to the deposition of a carbon layer over the carbon nodules. Finally, the P_CX_5.3 sample shows carbon nodules with sizes ranging from 30 to 70 nm, which is quite close to the CX values. The increase in the size of the nodules might be associated with a slight decrease in pH [52,74]. The presence of metal oxides does not seem to interfere with the formation of CX.

The SEM images suggest that the CX-containing samples would have a considerable porosity, so it is important to perform a proper characterization. However, another important issue is determining the concentration of carbon content in the composites, which can affect their final textural properties. For this purpose, thermogravimetric analyses were performed for the pristine CX and composites prepared via in situ methods. The results are shown in Figure S2. Generally, as was expected, metal oxide compounds catalyze CX combustion, with the onset of oxidation shifted towards lower temperatures. The carbon content of each sample, calculated from the TG curves, is shown in Table 1. For the CX_P-based composites, it can be observed that the concentration of the metal-based compounds is quite close to the target (20 wt.%). As expected, the CX_P_O_2_ sample contains a slightly higher quantity of metal-based compounds because of the partial burning of reactive carbon materials during the thermal treatment under air. Conversely, the P_CX-based composites do not display the desired concentration of metal oxide, i.e., they have a larger concentration of carbon material (~90 wt.%). This suggests that the presence of perovskite in the starting CX solution could affect the polymerization of resorcinol and formaldehyde, influencing the carbon yield during the formation of CX.

**Figure 4:**
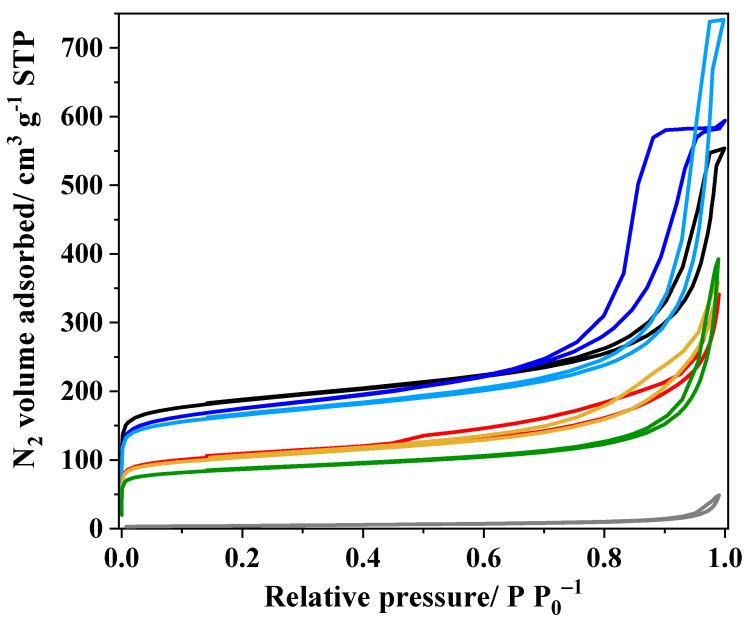
N_2_ adsorption isotherms at −196 °C for the pristine CX and P samples and the composites containing both compounds. (**▬**) P; (**▬**) CX; (**▬**) P + CX; (**▬**) CX_P_N_2_; (**▬**) CX_P_O_2_; (**▬**) P_CX_5.6; (**▬**) P_CX_5.3.

Concerning the textural properties, Figure 4 shows the N_2_ adsorption–desorption isotherms of all the samples. The P sample isotherm is typical of a non-porous material. Meanwhile, the CX sample displays a combination of types I and IV isotherms, indicative of micro-mesoporous materials according to the IUPAC recommendations [58]. The P_CX-based composites also have a similar isotherm profile, i.e., these are micro-mesoporous materials. Among these composites, the P_CX-5.6 sample has many mesopores, as indicated by the noticeable hysteresis loop. Meanwhile, the CX_P-based materials display a profile characteristic of a micro-macroporous solid, i.e., a combination of type I and type II isotherms [58].

Table 1 summarizes the textural parameters of the materials. As expected, the *S*_BET_ of the composites synthesized by supporting the metal oxides on CX via either in situ synthesis or physical mixing decreased by around 50 and 60%, respectively. This indicates that the concentration of carbon xerogel determines mainly the *S*_BET_ of the composites. Moreover, it cannot be discarded that perovskite nanoparticles may partially block the porosity of the carbon xerogel, slightly decreasing the *S*_BET_ values. In the case of the CX_P-based composites, the blockage of the porosity from CX is more evident than in the P + CX composite. It can be observed that the *S*_BET_ decreases more than 20%, as expected, because of the presence of metal-based compounds. This suggests an important blockage of CX pores by the metal-based compounds and the carbon materials, such as carbon nanotubes formed from the carbonization of the chelating agents. Interestingly, the carbon xerogel formed during the synthesis of the P_CX-based compounds developed a porosity similar to the CX sample. The presence of perovskite appears to increase the formation of mesopores, especially for the P_CX-5.6 sample, as evidenced by the difference in mesopore volume.

### 3.3. Surface Characterization

The electrochemical reactions occur at the interface between the surface of the materials and the electrolyte. Therefore, a proper characterization of the surface composition is essential to understand the behavior of the different materials towards the ORR. In that ambit, XPS spectroscopy was used to analyze the surface of the materials and determine the oxidation state of the cations and oxygen species that can participate in the ORR. Table S2 shows the mass percentage of the different elements analyzed. As expected, one observes a correlation between the C wt.% on the surface and the total bulk content determined by the TG analysis. Surprisingly, the CX_P-based materials show values close to that of P + CX, indicating the formation of small, well-dispersed metal compounds over the carbon xerogel. N was also detected for the P_CX-based materials, which might come from the decomposition of EDTA and nitrate precursors. In the case of P_CX-based composites, the concentration of the La^3+^, Mn^n+^, and Co^n+^ cations is rather low, which could affect their catalytic activity. This fact might be related to the formation of isolated and/or large particles that make the sample heterogeneous.

Figure S3 displays La 3d, Mn 2p, Co 2p, and O 1s core-level spectra for the pristine perovskite and the different composite materials. The La 3d spectrum of the pristine perovskite consists of two well-separated signals at around 833 eV (La 3d_5/2_) and 850 eV (La 3d_3/2_), which split into two contributions (Figure S3a) [54]. In the case of the composites, one observes a positive shift towards higher binding energies of around 1 eV and 1.8 eV for the P + CX sample and the composites prepared via in situ methods, respectively. Previous studies [14,18] observed that the positive shift emerges from the displacement of the electron cloud from the metallic cations to the lighter elements. Hence, this effect indicates an interaction between the metal oxide and carbon materials. As expected, the composites synthesized via in situ methods show a greater positive shift, suggesting stronger interactions between both phases. Regarding the spin-orbit splitting of the La 3d, the energy value of 16.8 eV suggests the presence of La^3+^ species [75,76], which agrees with the crystallite phases identified by XRD. The energy value of the spin-orbit splitting remains constant for all the different composites. However, the energy value of the multiplet splitting changes depending on the nature of the samples. For the samples containing the perovskite structure phase, the energy value is around 4.2 eV, whereas the energy value close to 3.8 eV indicates the presence of La(OH)_3_ species [76]. This suggests the formation of La(OH)_3_ structure also for the CX_P-based composites. Figure S3b depicts the spectra of Mn 2p with two asymmetrical peaks at about 642 eV and 653 eV, which are related to Mn 2p_3/2_ and Mn 2p_1/2_, respectively [54]. A positive shift is also observed in the main contribution, which suggests the interaction between both compounds. Regarding the spin-orbit splitting, the energy value of 11.6 eV indicates the presence of Mn^3+^ on the surface of the materials [54,77]. Meanwhile, the P_CX-based materials have a slightly higher energy value of 11.9 eV, which previous studies ascribed to MnO, confirming the XRD results [78]. Concerning the spectra of Co 2p, only the spectra of Co 2p for P, P + CX, and CX_P_O_2_ samples are shown in Figure S3c because the Co concentration on the surface for the other samples is below the detection limit. The spectrum is characteristic of two asymmetrical contributions at around 779 eV and 795 eV for Co 2p_3/2_ and Co 2p_1/2_, respectively [54,79]. Yet again, the positive shift related to the formation of interactions can be observed, especially for the CX_P_O_2_ sample. The spin-orbit splitting of 15.6 eV indicates the presence of Co^2+^ and Co^3+^ species in the samples [27]. Finally, Figure S3d displays the spectra of O 1s for the different samples where two different profiles can be distinguished. The pristine perovskite shows two peaks at 528.7 eV and 530.9 eV related to different oxygen species. Similarly to the metallic cations, the presence of CX also provokes a positive shift of 0.6 eV for both peaks in the P + CX samples. On the contrary, the composites synthesized through in situ methods display only one peak at 531.5 eV and 532 eV for CX_P-based and P_CX-based materials, respectively. Finally, Figure S4a shows the N 1s spectra of these composites, with a peak at around 399 eV.

**Figure 5:**
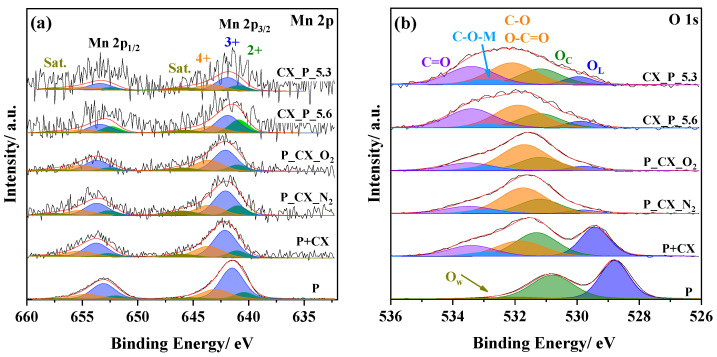
Deconvolution of the Mn 2p (**a**) and O 1s (**b**) of the composites.

Mn oxidation states and O species can determine the final performance in the ORR, so it is necessary to deconvolute the Mn 2p and O 1s spectra to quantify the contribution of the different species. Despite the fact that Co and N elements were not detected for all the materials, the Co 2p and N 1s were also deconvoluted to identify important catalytic sites that could make the difference. The results are depicted in Figure 5 and Figure S4. The Mn 2p_3/2_ and Mn 2p_1/2_ signals can be deconvoluted into three contributions related to different oxidation states (Figure 5a): Mn^2+^ (640.4–641.2 and 652.0–652.6 eV), Mn^3+^ (641.5–642.1 and 653.1–653.7 eV) and Mn^4+^ (643.0–643.6 and 654.6–655.2 eV) [17,77]. The higher binding energy value is related to Mn species in the composites. Moreover, a satellite peak can be distinguished at around 646 eV; this corresponds to the presence of Mn^2+^ species [77]. Figure 5b shows the O 1s spectrum for the P sample separated into three different peaks: lattice oxygen (O_L_) (528.8 eV), surface adsorbed oxygen species or hydroxyls (OH^−^) (O_C_) (530.8 eV), and adsorbed H_2_O molecules (O_W_) (532 eV) [15,16,80]. However, the presence of CX leads to the appearance of new peaks related to the carbon material. Therefore, the spectra are deconvoluted into five peaks related to O_L_ (529.6 eV), O_C_ (531.1 eV), carbonyl groups (C=O) (531.8 eV), C-O-M covalent bonds (533 eV), and carbon–oxygen ether-like single bonds (C-O) and carboxylic acid groups (O-C=O) (533.5 eV) [17,20,29,77]. The C-O-M covalent bonds (M = La, Mn, or Co) are associated with strong interactions between the metal oxide and CX.

Interestingly, as can be seen in Figure 5b, the presence of CX causes a positive shift towards higher binding energy values for the oxygen species (O_L_ and O_C_ species). This fact was previously ascribed to the strong interactions between the carbon material and metal oxide [14]. This interaction causes the displacement of the electron cloud from the metal cations to the lighter elements, increasing the binding energy [14,18]. In addition, the different metallic environments can also lead to changes in the binding energy of the oxygen species, such as the formation of La_2_O_3_ and La(OH)_3_ phases instead of the LaMnO_3_ phase [17].

The Co 2p_3/2_ and Co 2p_1/2_ for the perovskite-based materials can be separated into two peaks related to Co^2+^ (779.3–780.0 and 794.9–795.7 eV) and Co^3+^ (781.0–781.6 and 796.5–797.0 eV) (Figure S4b) [15,27,54]. On the contrary, the CX_P_O_2_ sample can be deconvoluted into three contributions corresponding to Co^0^ (779.3 and 794.3 eV), Co^2+^ (780.3 and 795.9 eV), and Co^3+^ (781.9 and 797.4 eV) [39,66]. Despite the formation of the Co_3_O_4_ phase, there are remaining Co^0^ species on the surface. The N 1s spectrum can be split into four peaks associated with pyridinic N (398.4 eV), Co-N species (399.3 eV), pyrrolic N (400.1 eV), and quaternary N (401.1 eV) (Figure S4c) [33,34,36]. Despite Qian et al. [33] concluded that the catalytic sites in ORR are Co-N species and not metallic Co particles or N-C species, Quílez-Bermejo et al. [81] observed that an increase in quaternary N species enhances the ORR significantly. Therefore, the positive effect on the catalytic activity of these species for the CX_P-based materials cannot be dismissed.

Table 2 shows atomic ratios obtained for A and B cations, different B cation oxidation states, and oxygen species. The B_Total_/A atomic ratio indicates that lanthanum segregation on the surface occurs in all composites, similar to previous studies [54,77]. For the composites where cobalt content was determinable, the Co/B_Total_ atomic ratio is very close to the nominal value. Regarding the Mn^4+^/Mn^3+^ ratio, it can be observed that the concentration of Mn^4+^ increases for the composites. Despite Mn^3+^ species being the desired species for ORR, a moderate concentration of Mn^4+^ species can enhance the ORR by favoring the surface hydroxide displacement step (O_2_^2−^/OH^−^), which is a rate-limiting step for ORRs in metal oxide-based materials [16,82]. Furthermore, the higher oxidative ability of Mn^4+^ can lead to the chemical disproportionation of peroxide into hydroxide and oxygen, enhancing the overall reaction [82]. Similar to Mn species, Co^3+^ species are required because this oxidation state has an optimal *e_g_* orbital filling that facilitates the O_2_^2−^/OH^−^ displacement [12]. Despite the decrease in the concentration of Co3+ species in composite materials, the catalytic activity of those sites is lower than that of Mn^3+^ species [11,12]. Therefore, the decrease should not have a significant effect on the catalytic activity. The quantity of chemisorbed oxygen (O_C_) species for the CX_P-based composites increases abruptly, especially for the CX_P_N_2_ material. Previous studies [16,77,82] reported that O_C_ species are mainly associated with oxygen vacancies, which can enhance the ORR through the improvement of electrical conductivity and the mobility of the oxygen ions. Moreover, they can facilitate the O_2_^2−^/OH^−^ displacement, which directly influences the electrochemical reaction [16,82]. Another important oxygen species is the C-O-M interaction bonds that can enhance the electron transfer and, remarkably, the O_2_^2−^/OH^−^ displacement during ORRs [20,29,32]. As expected, the in situ methods generated a higher concentration of C-O-M interaction bonds. The slightly lower concentration of CX_P_O_2_ material might result from the oxidation of the metal cations (Mn^n+^ and Co^n+^) during the thermal treatment under air, which could decrease these interactions. For the CX_P-based materials, it can be observed that the concentration of Co-N_x_ species increases slightly after the thermal treatment in the air. On the contrary, the N wt.% decreases, which suggests that the Co-N_x_ are more stable than the other N species. The presence of Co-N_x_-C species can incur a difference in the ORR activity as these were claimed to strongly affect the activity instead of Co particles. These groups can optimize the chemisorption of intermediates and facilitate electron transfer [34].

### 3.4. Electrochemical Characterization

**Figure 6:**
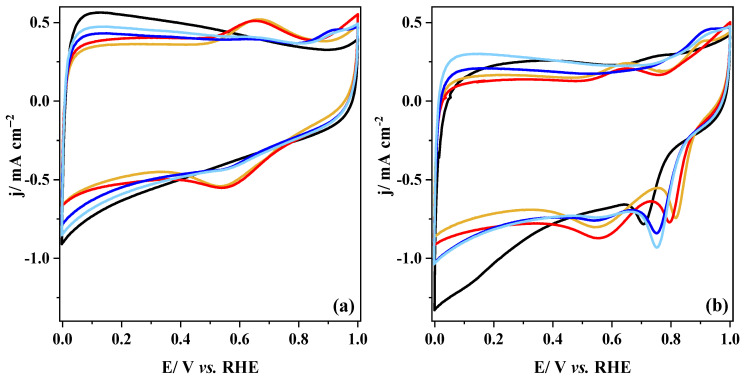
Cyclic voltammetry of the composites in 0.1 M KOH medium saturated with either N_2_ (**a**) or O_2_ (**b**). Scan rate: 10 mV s^−1^. (**▬**) CX; (**▬**) CX_P_N_2_; (**▬**) CX_P_O_2_; (**▬**) P_CX_5.6; (**▬**) P_CX_5.3. All current densities are reported by the geometric area of the electrode.

As previously mentioned, the method of synthesis used to produce composites can significantly impact their physicochemical properties. Thereby, the electrochemical properties of the as-prepared composites are expected to be affected as well; in particular, the performance of the ORR reaction could be impacted. To check this, the composites were electrochemically characterized by cyclic voltammetry in 0.1 M KOH solution saturated with either N_2_ or O_2_. The obtained results are displayed in Figure 6 and Figure S5. The pristine CX and metal oxide materials were also characterized alone in the same conditions to highlight the synergistic effect in composites. In the N_2_-saturated medium (Figure S5a), as expected, the pure perovskite exhibits the lowest voltammetric charge; additionally, the absence of redox processes agrees with the poor electrical conductivity and low surface area of the material. CX shows a CV profile common to carbon materials, with a double-layer capacitance only related to surface charging processes (Figure 6a). In the case of the composites, it can be observed that the double-layer capacitance is quite correlated to the specific surface area value, *S*_BET_ (Table 1). This value depends mainly on the carbon content since it has a larger *S*_BET_ than metal-based compounds, which influences the final surface area of the composites. The sample P + CX has the lowest double-layer capacitance, but it displays pseudocapacitive contributions related to the Mn redox processes (Figure S5a). In the anodic scan, one can observe the oxidation of Mn^2+^ species to Mn^3+^ species at around 0.65 V vs. RHE. Meanwhile, in the cathodic scan, the reverse reaction, i.e., the reduction of Mn^3+^ species to Mn^2+^ species, occurs at around 0.55 V vs. RHE [14,77,83]. Interestingly, despite the fact that the sample contains no perovskite structure, the CX_P-based materials show the Mn^2+^/Mn^3+^ redox processes at the same potentials as the P + CX sample (Figure 6a). This strongly suggests that the method used can develop Mn-based oxides with various Mn oxidation states on the surface similar to the perovskite surface. Additionally, the larger current contributions from the redox processes indicate a significant interaction between both compounds, favoring charge transfer. The small particle size of the metal-based compounds might favor the interaction with CX. The low crystallinity of these particles could also provide a higher number of accessible active sites [17]. Moreover, a starting anodic current can be discerned at around 0.9 V vs. RHE, which is ascribed to the Co^2+^/Co^3+^ redox oxidation process, indicating the presence of Co species on the surface [79].

On the contrary, the P_CX-based composites show the poor contribution of the Mn^2+^/Mn^3+^ redox processes, which might be explained by the lower concentration of Mn cations on the surface (Figure 6a). However, previous authors also reported similar poor Mn redox processes for MnO-based composites [37,38]. Hence, it cannot be discarded that large particles could be formed, leading to the formation of large domains of MnO species. Apart from the poor Mn^2+^/Mn^3+^ redox processes, a small anodic peak at around 0.9 V vs. RHE can be noticed related to the formation of MnOOH species and with the Co^2+^/Co^3+^ redox oxidation process [77,79].

Meanwhile, the CVs in the O_2_-saturated medium show that all materials exhibit activity towards ORR (Figure 6b and Figure S5b). Indeed, the voltammograms display a cathodic peak in the range of 0.7–0.85 V vs. RHE related to oxygen reduction for CX-containing samples. As expected, the process occurs at a lower potential (~0.6 V vs. RHE) in the case of pure metal oxide (Figure S5b), which highlights the importance of carbon materials in the final ORR activity of the material. Regarding the synthesis methods for the composites, in situ methods provide materials with a greater onset potential, especially the CX-P-based composites. The ORR performance will be discussed in detail later by conducting linear sweep voltammetry (LSV) experiments.

### 3.5. Catalytic Activity Towards ORR

**Figure 7:**
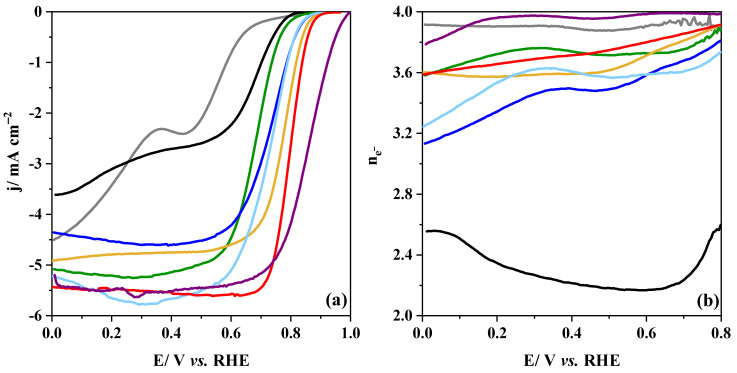
(**a**) RDE linear sweep voltammograms for composites in 0.1 M KOH saturated with O_2_ at 1600 rpm; (**b**) number of electrons involved in ORR at increasing potential. (▬) P; (**▬**) CX; (**▬**) P + CX; (**▬**) CX_P_N_2_; (**▬**) CX_P_O_2_; (**▬**) P_CX_5.6; (**▬**) P_CX_5.3; (▬) 20 wt.% Pt/C. All current densities are reported by the geometric area of the electrode.

The electrocatalytic activity of pristine and composite materials was assessed by polarization curves using an RRDE at 1600 rpm in 0.1 M KOH saturated with O_2_. Figure 7 shows the LSV curves for all the samples.

The P sample exhibits a double-plateau profile, which is consistent with previous results [14]. The first region (0.7–0.5 V vs. RHE) is associated with the direct oxygen reduction reaction to hydroxide, and the second region (0.3–0.1 V vs. RHE) is associated with the reduction of remaining oxygen and hydrogen peroxide to hydroxide (Figure 7a). In contrast, the CX sample shows better electrocatalytic activity, which proves that carbon-based materials could also be catalytically active for the ORR. Among the composites, the P + CX sample displays the lowest onset potential, which might be related to the weak interactions between both materials when using physical mixing. Apart from the formation of C-O-M bond species, in situ approaches also favor the formation of highly active sites; this results in a lower onset potential, especially in the case of the CX-P-based composites.

Figure 7b depicts the number of electrons transferred during the ORR. The reaction can proceed via two mechanisms: the 2-electron pathway and the 4-electron pathway [25]. The 2-electron pathway consists of reducing the O_2_ to HO_2_^−^, which can be further reduced to OH^−^. Meanwhile, the 4-electron pathway involves the direct reduction of O_2_ to OH^−^. This pathway is preferable because it is the most efficient and avoids the production of corrosive peroxide species. Previous studies [12,32,38] proposed that the 4-electron pathway in metal oxide-based materials can proceed via four steps through the interaction of surface *3d* metal transition cation with reactants and products: (i) surface hydroxide displacement, (ii) surface peroxide formation, (iii) surface oxide formation and (iv) surface hydroxide regeneration. The former and latter steps are the rate-determining steps of the reaction, which are related to the O_2_^2−^/OH^−^ displacement [12].

The metal oxide exhibits a direct reduction via the 4-electron pathway, whereas the pure carbon material is close to the 2-electron pathway. In the case of the composite materials, the ORR proceeds through a pathway quite similar to perovskite, indicating that the influence of the metal oxide is stronger than that of the carbon material for the overall ORR mechanism. A Pt/C commercial catalyst was also characterized under the same conditions as a reference material for ORR. As expected, the material features great catalytic activity and selectivity for a 4-electron pathway.

Table 3 summarises the electrochemical parameters obtained from Figure 7. The ORR in the perovskite material proceeds via a 4-electron pathway (n_e−_ = 3.93) in agreement with the above-mentioned mechanisms. However, its poor electrical conductivity and surface area limit its performance in terms of onset potential (0.78 V vs. RHE) and limiting current density (−2.36 mA cm^−2^). The pristine carbon material exhibits a better ORR performance than the pristine metal oxide but with a 2-electron pathway (n_e−_ = 2.23). Meanwhile, mixing both pristine materials resulted in a sample with better ORR performance by overcoming the limitations of the metal oxide. Moreover, the carbon material can act as a co-catalyst, providing HO_2_^−^ species to the adjacent metal oxide active sites for their further reduction to OH^−^ [24,25]. An increase in the Mn^4+^, O_C_, and C-O-M bond species can also be detected (Table 2), which probably enhances electron transfer and facilitates the O_2_^2−^/OH^−^ displacement, improving the overall ORR reaction [29,32,82].

In general, it can be observed that the ORR activity of the composites is still far from the Pt/C electrocatalytic performance in terms of onset potential and selectivity to a 4-electron pathway. However, the composites prepared via in situ methods exhibit an outstanding ORR activity compared with those obtained by the physical mixing method. The higher concentration of O_C_ and C-O-M bond species can facilitate the electron transfer, but this is not the only reason. Among the CX_P-based materials, the CX_P_N_2_ sample exhibits the highest ORR performance, which might be ascribed to the formation of heterointerfaces of MnO/Co. Those interfaces were reported to promote charge transfer and provide extra electrons for the reaction [42]. It was described that the presence of metallic Co improves the electrical conductivity, whereas the MnO facilitates the adsorption of the intermediate species, hence decreasing the ORR energy barrier [40]. The Co-N_x_-C species can also contribute positively to the ORR. These species can optimize the chemisorption of intermediates, facilitate electron transfer, and reduce the energy barrier [34,35]. Moreover, the positive catalytic activity of the quaternary N groups cannot be discarded. On the contrary, the CX_P_O_2_ sample exhibits a loss of activity, especially in terms of limiting current density, which drops to −4.76 mA cm^−2^ (vs. −5.56 mA cm^−2^ for the CX_P_N_2_ sample). This might be linked to the formation of the Co_3_O_4_ crystal phase, which decreases the number of MnO/Co heterointerfaces. The decrease in O_C_ and C-O-M bond species might also negatively affect the catalytic performance. Despite the decrease in the number of active sites, the catalytic activity does not drop abruptly, indicating that the Co-N_x_-C species are highly active sites for ORR. Thus, these active sites can produce a synergistic effect with the MnO/Co heterointerfaces, which results in an excellent performance. Apart from the above-mentioned phenomena, the low crystallinity of the metal compounds can also favor the performance of these composites because of the formation of new active sites from the lattice defects. All these catalytic sites existing in the CX_P_N2 sample are shown in Figure S7.

Among the composites prepared via in situ methods, the P_CX-based composites show a lower ORR performance than the CX_P-based composites. This might be mainly because the low concentration of the metal cations on the surface decreases the number of the MnO/Co heterointerfaces, which are important active sites. Furthermore, the absence of the Co-N_x_-C species confirms the importance of these species for the activity towards ORR. The composites exhibit quite similar behavior with differences in the limiting current density only: −4.58 mA cm^−2^ for P_CX_5.6 vs. −5.67 mA cm^−2^ for P_CX_5.3. Despite the slightly lower concentration of metal compound at the surface (Table S2), sample P_CX_5.3 displays the same onset potential as P_CX_5.6 samples (0.85 V vs. RHE). This might be ascribed to a higher content of Mn^n+^ in comparison to other metal cations on the surface and to a moderate Mn^4+^/Mn^3+^ ratio (Table 2). Moreover, according to the XRD study, the higher concentration of the La_2_O_3_ crystal phase provides more C-O-La bond sites that promote electron transfer and facilitate the O_2_^2−^/OH^−^ displacement, which in turn accelerates the overall reaction [32].

The Tafel slope value provides information about the kinetics of electron transfer involved in the ORR and determines the rate-determining step (Figure S6). In this context, values near 120 mV dec^−1^ are related to the first electron transfer process, whereas values close to 60 mV dec^−1^ suggest that the rate-determining step is the protonation of superoxide to form peroxide [84]. These values are close to those observed for the pristine perovskite and CX samples, respectively. The poor electrical conductivity of the metal oxide hinders the first electron transfer process since electrons are required from the solid for it to occur. This explains the high value of the Tafel slope observed in sample P (160 mV dec^−1^). Meanwhile, the value of the P + CX sample (80 mV dec^−1^) agrees with its composition, i.e., this value indicates the presence of sites with either the first electron transfer process or the protonation of superoxide as a rate-determining step. In the case of the composites prepared via in situ methods, faster kinetics with Tafel slope values in the range 47–57 mV dec^−1^ are obtained, indicating that the rate-determining step is the protonation of superoxide to form peroxide [84]. The great kinetics might be mainly associated with the presence of Co/MnO heterointerfaces, C-O-M bond species, and oxygen vacancies that favor electron transfer. The better kinetics of the CX_P-based materials might be ascribed to the presence of the Co-N_x_-C sites, as well as to the higher concentration of metal-based compounds on the surface.

## 4. Conclusions

Two different synthesis methods have been developed to prepare composites of LaMn_0.7_Co_0.3_O_3_ perovskite over carbon xerogel. On the one hand, the metal oxide was synthesized over a pre-existing carbon xerogel while, on the other hand, the carbon xerogel was prepared in the presence of the metal oxide and put in suspension in the gel precursor solution. The as-prepared materials were targeted as catalysts for oxygen reduction reactions (ORRs) in an alkaline medium. However, both routes were unsuccessful in preparing the composites because either the perovskite structure was not formed within the carbon structure or because it was destroyed upon pyrolysis to form the carbon support, respectively. Despite this fact, the goal of enhancing the interaction between both compounds was achieved by forming C-O-M bond species, which improved electron transfer. The increase in the concentration of chemisorbed oxygen species also had a positive impact on electron transfer and facilitated the O_2_^2−^/OH^−^ displacement. However, the formation of MnO/Co heterointerfaces strongly enhanced the ORR performance because of better electron transfer and improvement of the adsorption of intermediates.

Among the different composites, those prepared by synthesizing the metal oxide in the presence of the carbon xerogel display a better ORR performance than composites prepared via the opposite method. This can be ascribed to the higher concentration of metal compounds such as MnO, Co, and La_2_O_3_ on the surface. Moreover, the small particle sizes of these materials and their good distribution can promote the interaction between both phases. The low crystallinity of the metal compounds can also lead to higher concentrations of active sites from the lattice defects. Thus, these composites have high concentrations of active sites, including C-O-M covalent bond species, MnO/Co heterointerfaces, and lattice defects that enhance the ORR. Apart from the above-mentioned sites, these composites also contain Co-N_x_-C species that are highly active sites for ORRs. Furthermore, the synergistic effect of MnO/Co heterointerfaces and Co-N_x_-C species that may promote even higher catalytic activity cannot be discarded. It has also been demonstrated that subjecting this composite to thermal treatment under air decreases the electrocatalytic activity due to a change in the Co phase. This crystal phase is important for the formation of MnO/Co heterointerfaces.

## Figures and Tables

**Table 1:** Carbon percent obtained from the TG experiments, and textural parameters obtained from the N_2_ adsorption isotherms.

Sample	Carbon Content ^a^	*S*_BET_ ^b^	*V*_DR_ ^c^	*V*_meso_ ^d^	*V*_Total_ ^e^
	wt.%	(m^2^ g^−1^)	(cm^3^ g^−1^)	(cm^3^ g^−1^)	(cm^3^ g^−1^)
**P**	0	15	0.01	0.02	0.03
**CX**	100	710	0.28	0.28	0.56
**P + CX**	50	325	0.13	0.18	0.31
**CX_P-N_2_**	82	400	0.17	0.19	0.36
**CX_P-O_2_**	80	390	0.16	0.24	0.40
**P_CX-5.6**	88	650	0.27	0.60	0.87
**P_CX-5.3**	89	620	0.26	0.36	0.62

^a^ Determined from the TG curves performed under air. ^b^ *S*_BET_: specific surface area calculated by the BET method. ^c^ *V_DR_*: micropore volume calculated by the Dubinin–Radushkevich equation. ^d^ *V*_meso_: mesopore volume calculated by subtracting the micropore volume from the total volume. ^e^ *V*_Total_: total volume determined at P/P^0^ = 0.95, i.e., macropores excluded.

**Table 2:** Experimental atomic ratios of the cations of the samples and deconvolution data obtained from Figure 2.

Sample	B_Total_/A ^a^	Co/B_Total_^b^	Mn^4+^/Mn^3+^	Co^3+^/Co^2+^	O_C_/O_L_^c^	O_C-O-M_/O_L_^d^	Co-N_x_/N_Total_^e^
**P**	0.62	0.24	0.37	1.97	0.90	0.00	-
**P + CX**	0.55	0.24	0.48	1.24	1.20	0.21	-
**CX_P_N_2_**	0.25	-	0.50	-	5.80	1.67	0.15
**CX_P_O_2_**	0.41	0.30	0.63	1.54	5.30	1.40	0.19
**P_CX_5.6**	0.59	-	0.45	-	3.00	1.65	-
**P_CX_5.3**	0.69	-	0.53	-	2.70	1.64	-

^a^ B_Total_/A: B_Total_ is the sum of Mn^n+^ and Co^n+^ cations and A stands for La^3+^ cations. ^b^ Co/B_Total_: Co is the concentration of Co^n+^ cations. ^c^ O_C_/O_L_: O_C_ represents the chemisorbed oxygen species, whereas O_L_ is the lattice oxygen species. ^d^ O_C-O-M_/O_L_: O_C-O-M_ species are created by interacting strongly with metal cations and carbon through oxygen. ^e^ Co-N_x_/N_Total_: Co-N_x_ represents the N species bonded to Co^n+^ cations, and N_Total_ represents all N detected by XPS.

**Table 3:** Onset potential, number of transferred electrons, limiting current density, and Tafel slope obtained from the polarization curves of the composites.

Sample	$E_{onset}$ ^a^	$n_{e^{-}}$ ^b^	$j_{lim}$ ^c^	Tafel Slope ^d^
	V vs. RHE	-	mA cm^−2^	mV dec^−1^
**P**	0.78	3.93	−2.36	160
**CX**	0.79	2.23	−2.70	60
**P + CX**	0.82	3.75	−5.14	80
**CX_P_N_2_**	0.89	3.86	−5.56	47
**CX_P_O_2_**	0.87	3.81	−4.76	53
**P_CX_5.6**	0.85	3.68	−4.58	57
**P_CX_5.3**	0.85	3.61	−5.67	57
**Pt/C**	0.98	3.98	−5.51	60

^a^ E_onset_: onset potential determined at −0.1 mA cm^−2^. ^b^ n_e_^−^: number of electrons, calculated at 0.7 V vs. RHE. ^c^ j_lim_: limiting current density, determined at 0.4 V vs. RHE. ^d^ Tafel slope: values calculated in the kinetics-limited region.

## Data Availability

Data are contained within the article and Supplementary Materials.

## Supplementary Materials

The following supporting information can be downloaded at: https://www.mdpi.com/article/10.3390/nano14161362/s1, Figure S1: X-ray diffraction patterns for (a) P_CX-based composites and (b) CX_P-based composites; Figure S2: TG curves for the carbon-containing samples. (**▬**) CX; (**▬**) CX_P_N_2_; (**▬**) CX_P_O_2_; (**▬**) P_CX_5.6; (**▬**) P_CX_5.3; Figure S3: XPS spectra obtained from (a) La 3d; (b) Mn 2p; (c) Co 2p; and (d) O 1s for the pristine perovskite and composite materials. (**▬**) P; (**▬**) P + CX_;_ (**▬**) CX_P_N_2_; (**▬**) CX_P_O_2_; (**▬**) P_CX_5.6; (**▬**) P_CX_5.3; Figure S4: XPS spectra and deconvoluted XPS spectra at (a) N 1s; (b) Co 2p; and (c) N 1s for the different materials; Figure S5: Cyclic voltammetry of the composites in 0.1 M KOH medium saturated with either N_2_ (a) or O_2_ (b). Scan rate: 10 mV s^−1^. (**▬**) P and (**▬**) P + CX. All current densities are reported by the geometric area of the electrode; Figure S6: RDE linear sweep voltammograms for composites in 0.1 M KOH saturated with O_2_ at 1600 rpm; (b) number of electrons involved in ORR at increasing potential. (**▬■▬**) P; (**▬■▬**) CX; (**▬♦▬**) P + CX; (**▬▼▬**) CX_P_N_2_; (**▬▲▬**) CX_P_O_2_; (**▬◄▬**) P_CX_5.6; (**▬►▬**) P_CX_5.3; (▬●▬) 20 wt.% Pt/C. Figure S7: Scheme of the different active sites involved in the oxygen reduction reaction for the CX_P_N_2_ sample. The balls with different colours indicate the different crystal phases: (●) La_2_O_3_; (●) La(OH)_3_; (●) MnO; and (●) Co. Table S1: Crystallite size determined by the Scherrer equation; Table S2: The mass percent of the different elements analyzed by XPS.
