# Supplementary material for: Development of In Situ Methods for Preparing La-Mn-Co-Based Compounds over Carbon Xerogel for Oxygen Reduction Reaction in an Alkaline Medium
Source: Nanomaterials (Basel). 2024 Aug 19;14(16):1362. doi: 10.3390/nano14161362 (PMC11356951; doi:10.3390/nano14161362)
Supplement: Supplementary file 1 [file nanomaterials-14-01362-s001.zip › nanomaterials-3163694-supplementary.pdf]

Supporting Information

Development of in situ methods for preparing La-Mn-Co-based compounds over carbon xerogel for oxygen reduction reaction in an alkaline medium

Jhony Xavier. Flores-Lasluisa[1], Bryan Carré[1], Joachim Caucheteux[1], Philippe Compère[2], Alexandre F. Léonard[3], Nathalie Job[1]

[1]Department of Chemical Engineering - NCE (Nanomaterials, Catalysis, Electrochemistry), University of Liège, B6a, Allée du six août 13, B-4000 Liège, Belgium
[2]Functional and Evolutive Morphology, UR FOCUS, and Center for Applied Research and Education in Microscopy (CAREM-ULiege), University of Liège, 4000 Liège, Belgium
[3]Department of Chemical Engineering - CARPOR, University of Liège, B6a, Allée du six août 13, B-4000 Liège, Belgium

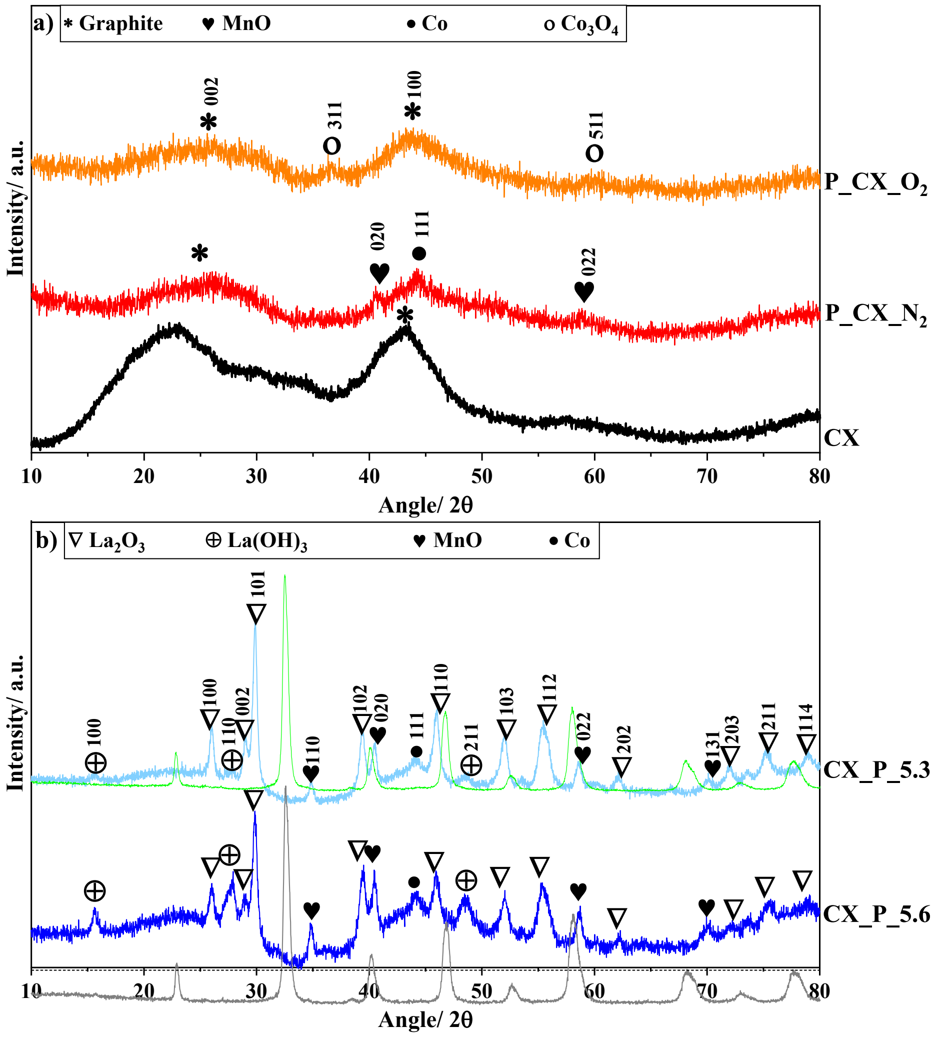

Figure S.1. X-ray diffraction patterns for (a) P_CX-based composites and (b) CX_P-based composites.

To determine the crystallite size of the different crystal phases, the Scherrer equation (Eq. S.1.) was applied to the most intense peaks and isolated to avoid miscalculation. In the case of the $LaMnO_3$ phase, the peak at 2θ value close to 23° was selected, whereas for the $La(OH)_3$ and La_2O_3 phases were selected the peaks at 2θ values close to 16 and 30°, respectively. Regarding the MnO and Co phases, it was taken the peaks at 2θ values close to 35 and 44°, respectively.

The Scherrer equation is the following one [1]:

$$D_c = \frac{k\lambda}{\beta\cos(\theta)} \qquad \text{Eq. S.1.}$$

where *Dc* is the crystallite size (nm); *k* is the constant related to the grain shape whose value is 0.89; *λ* is the wavelength of the radiation source which is 0.15406 nm; *β* is the full width at half maximum (FWHM) in radians, and *θ* is the Bragg angle.

Table S.1. Crystallite size determined by the Scherrer equation.

**Sample**	**C**	**$LaMnO_3$**	**La_2O_3**	**$La(OH)_3$**	**MnO**	**Co**	**Co_3O_4**
	nm	nm	nm	nm	nm	nm	nm
**P**	-	25	-	-	-	-	-
**P+CX**	-	24	-	-	-	-	-
**CX-P-N_2**	*	-	-	-	*	*	-
**CX-P-O_2**	*	-	-	-	-	-	*
**P-CX-5.6**	*	-	17	14	22	8	-
**P-CX-5.3**	*	-	18	10	18	11	-

(-) Indicates the non-presence of the crystal phase in the composite.

(*) Indicates the presence of the crystal phase, but the determination of the crystallite size was not possible.

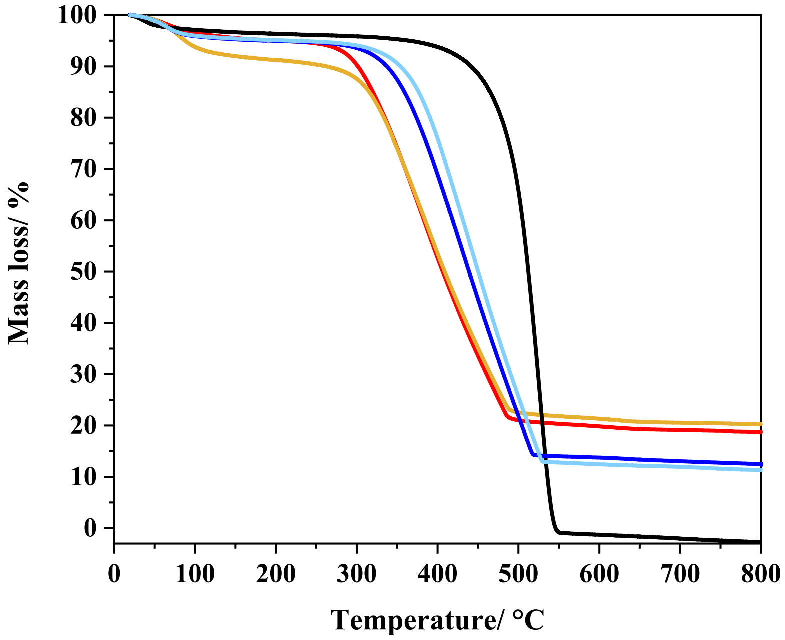

Figure S.2. TG curves for the carbon-containing samples. (—) CX; (—) CX_P_N_2; (—) CX_P_O_2; (—) P_CX_5.6; (—) P_CX_5.3.

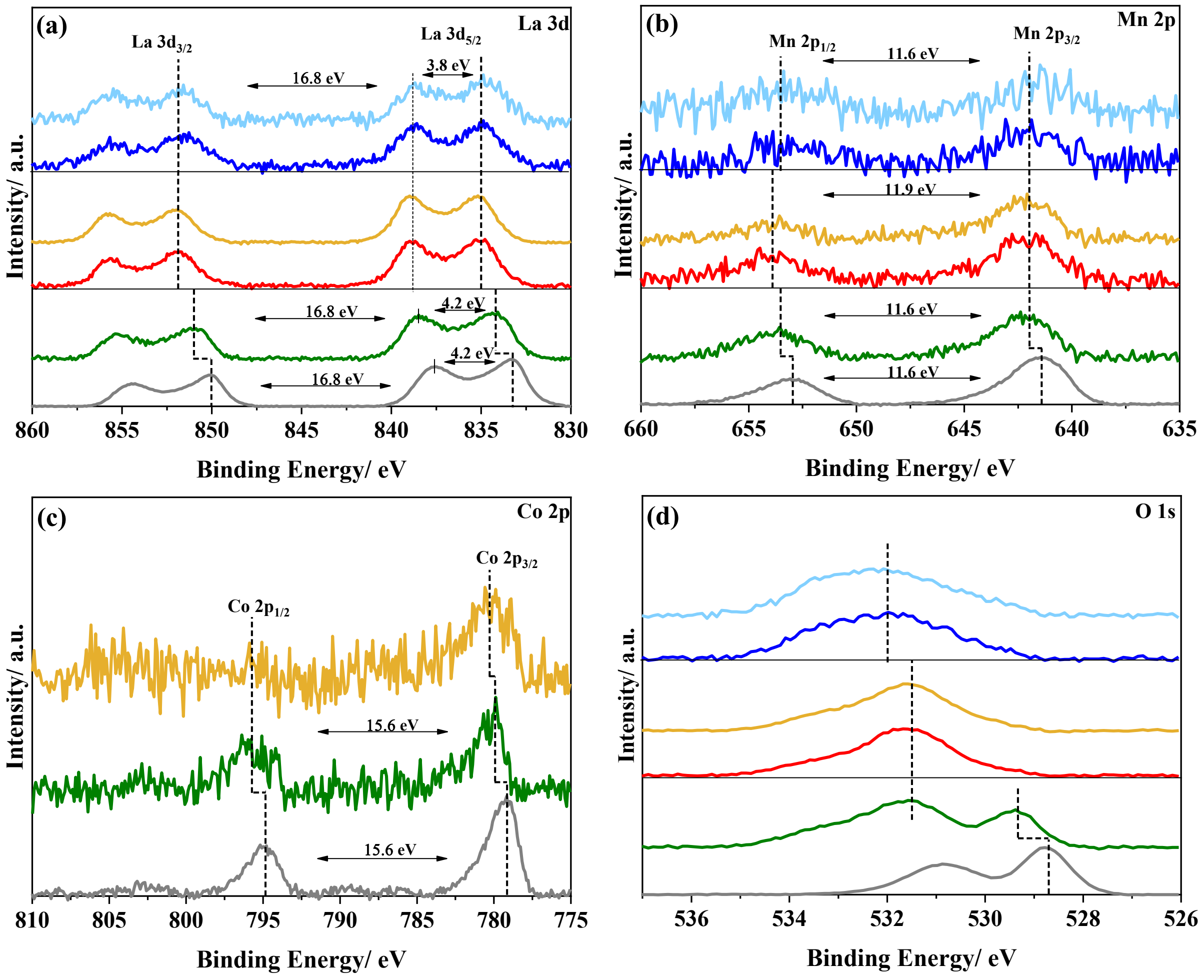

Figure S.3. XPS spectra obtained from (a) La 3d; (b) Mn 2p; (c) Co 2p; and (d) O 1s for the pristine perovskite and composite materials. (▬) P; (▬) P+CX; (▬) CX_P_N_2; (▬) CX_P_O_2; (▬) P_CX_5.6; (▬) P_CX_5.3.

Table S.2. The mass percent of the different elements analyzed by XPS.

Sample	C	O	N	La	Mn	Co
	wt.%	wt.%	wt.%	wt.%	wt.%	wt.%
**P**	13.85	22.77	-	50.67	9.49	3.21
**P+CX**	73.94	9.70	-	13.41	2.21	0.74
**CX-P-N_2**	78.79	7.57	1.58	10.98	1.08	-
**CX-P-O_2**	74.74	10.07	1.23	11.97	1.36	0.62
**P-CX-5.6**	93.29	3.66	-	2.47	0.58	-
**P-CX-5.3**	94.59	3.54	-	1.47	0.40	-

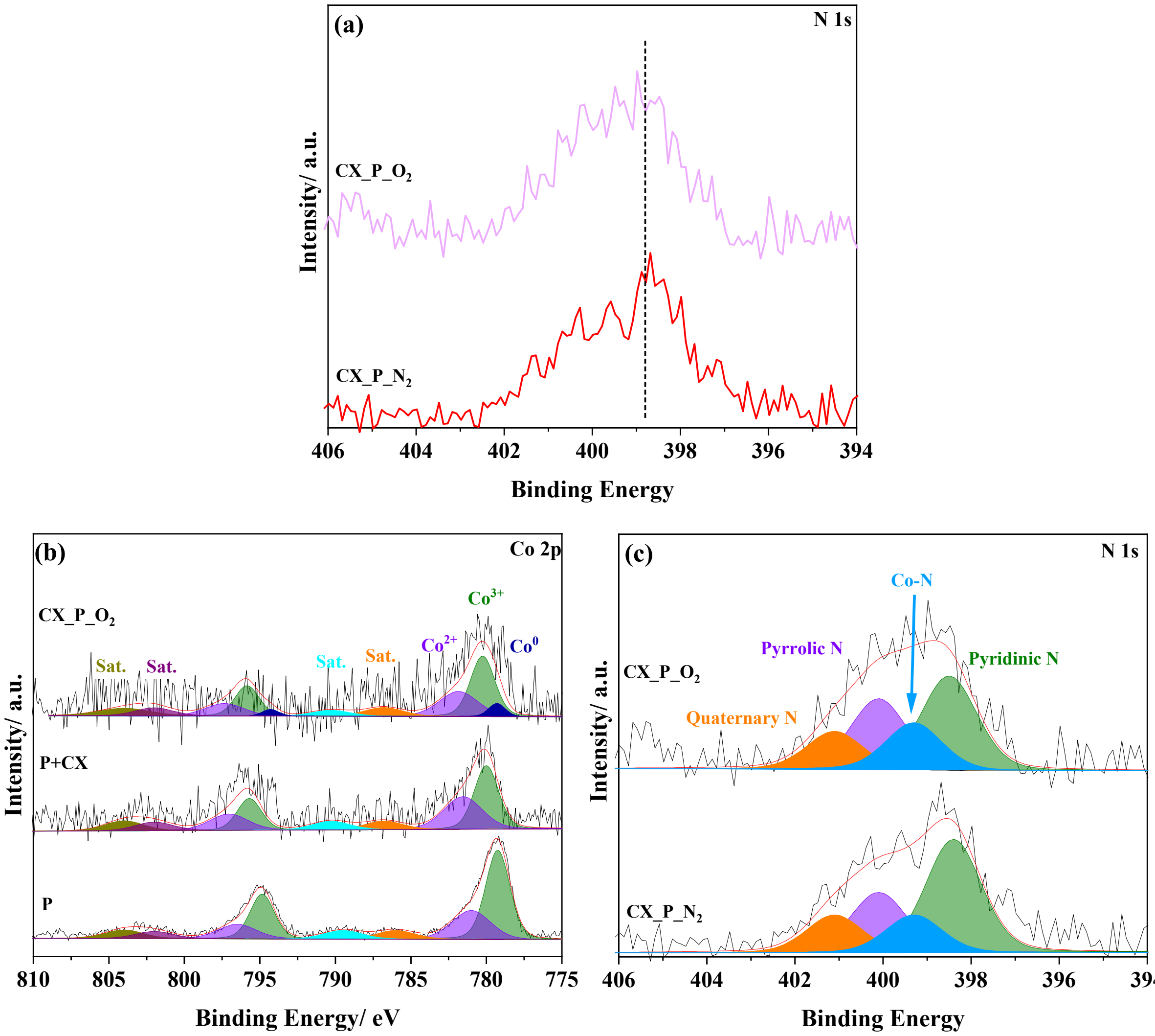

Figure S.4. XPS spectra and deconvoluted XPS spectra at (a) N 1s; (b) Co 2p; and (c) N 1s for the different materials.

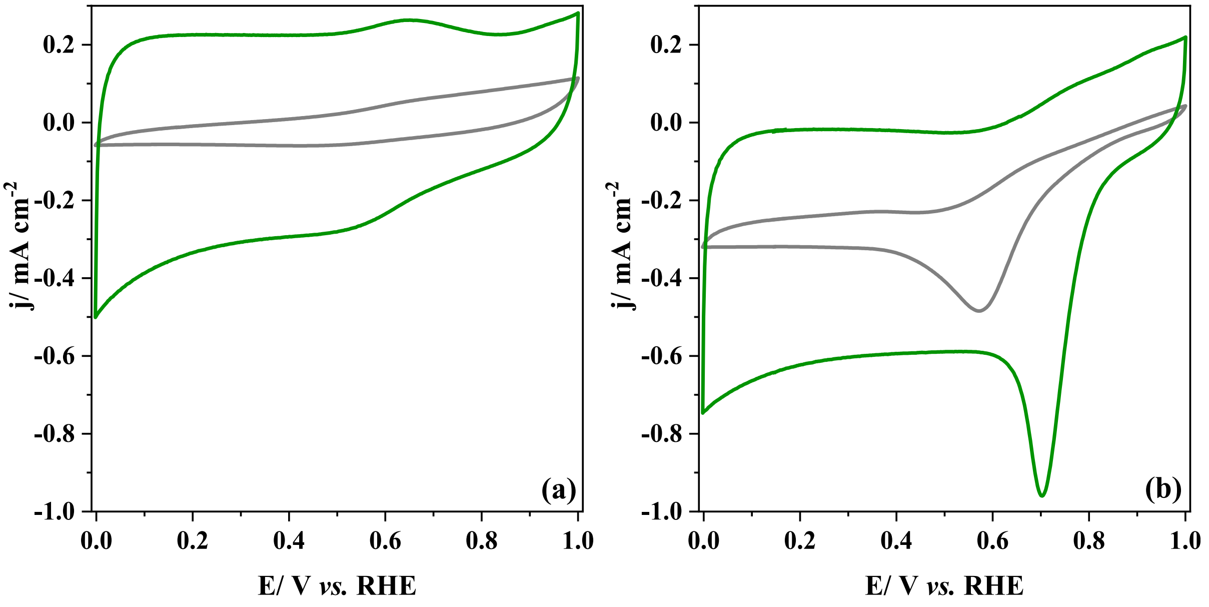

Figure S.5. Cyclic voltammetry of the composites in 0.1 M KOH medium saturated with either N_2 (a) or O_2 (b). Scan rate: 10 mV s^{-1}. (▬) P and (▬) P+CX. All current densities are reported by the geometric area of the electrode.

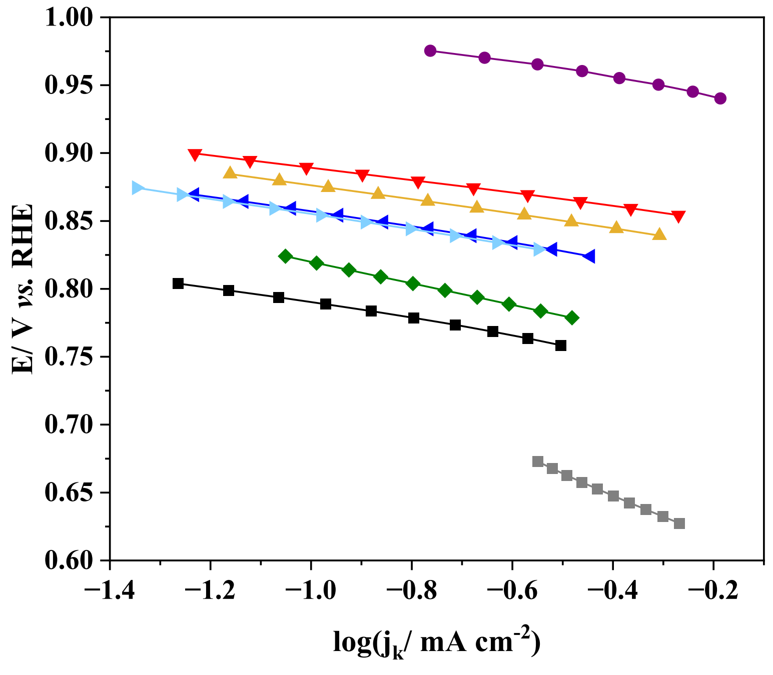

Figure S.6. RDE linear sweep voltammograms for composites in 0.1 M KOH saturated with O_2 at 1600 rpm; (b) number of electrons involved in ORR at increasing potential. (▬■▬) P; (▬■▬) CX; (▬◆▬) P+CX; (▬▼▬) CX_P_N$_2$; (▬▲▬) CX_P_O$_2$; (▬◀▬) P_CX_5.6; (▬▶▬) P_CX_5.3; (▬●▬) 20 wt.% Pt/C.

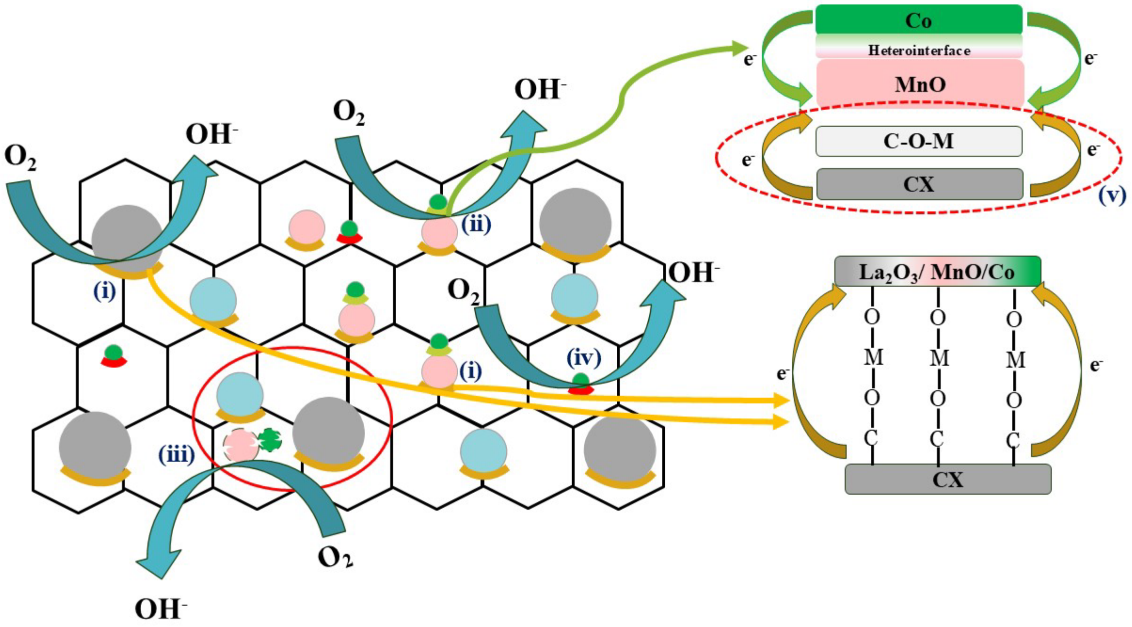

Figure S.7. Scheme of the different active sites involved in the oxygen reduction reaction for the CX_P_N_2 sample. The balls with different colours indicate the different crystal phases: (●) La_2O_3; (●) $La(OH)_3$; (●) MnO; and (●) Co.

Firstly, it can be determined that at least five different active sites catalyze the ORR, and a synergistic effect due to the presence of these sites may further enhance the ORR performance.

The C-O-M covalent bonds can lead to great ORR performance due to the movement of electrons from the carbon to the metal-based compounds through these covalent bonds (case i) [2-4]. This fact facilitates the the O_2^{2-}/OH^- displacement [3,4]. Similarly, the Co/MnO heterointerfaces favour the adsorption of oxygen species due to the movement of electrons from the Co phase to the MnO phase (case ii). It cannot be discarded that also electrons could migrate from the carbon material to the MnO facilitating even more the ORR (case v) [5].

In addition, the Mn, Co-based materials with low crystallinity stabilized by La-based compounds generate active sites from lattice defects (case iii) [6]. Finally, the Co-Nx-C sites can also promote the ORR by favouring the chemisorption of intermediates and facilitating electron transfer (case iv) [7].

References

[1] Y. Waseda, E. Matsubara, K. Shinoda, X-Ray Diffraction Crystallography, Springer Berlin Heidelberg, Berlin, Heidelberg, 2011. https://doi.org/10.1007/978-3-642-16635-8.

[2] V. Mishra, B.D. Mohapatra, T. Kumar, G.G. Ranga, Effect of MnO Content on the Oxygen Reduction Activity of MnO / C Nanostructures, (2023). https://doi.org/10.1007/s12678-023-00836-9.

[3] N. Wang, J. Liu, W. Gu, Y. Song, F. Wang, Toward synergy of carbon and La2O3 in their hybrid as an efficient catalyst for the oxygen reduction reaction, RSC Adv 6 (2016) 77786–77795. https://doi.org/10.1039/c6ra17104d.

[4] T. Li, J. Liu, X. Jin, F. Wang, Y. Song, Composition-dependent electro-catalytic activities of covalent carbon-LaMnO3 hybrids as synergistic catalysts for oxygen reduction reaction, Electrochim Acta 198 (2016) 115–126. https://doi.org/10.1016/j.electacta.2016.02.027.

[5] L. Wang, J. Huang, X. Hu, Z. Huang, M. Gao, D. Yao, T. Taylor Isimjan, X. Yang, Synergistic vacancy engineering of Co/MnO@NC catalyst for superior oxygen reduction reaction in liquid/solid zinc-air batteries, J Colloid Interface Sci 660 (2024) 989–996. https://doi.org/10.1016/j.jcis.2024.01.143.

[6] M. García-Rodríguez, D. Cazorla-Amorós, E. Morallón, Enhanced lanthanum-stabilized low crystallinity metal oxide electrocatalysts with superior activity for oxygen reactions, Electrochim Acta 479 (2024) 143858. https://doi.org/10.1016/j.electacta.2024.143858.

[7] C. Tang, B. Wang, H.F. Wang, Q. Zhang, Defect Engineering toward Atomic Co–Nx–C in Hierarchical Graphene for Rechargeable Flexible Solid Zn-Air Batteries, Advanced Materials 29 (2017) 1–7. https://doi.org/10.1002/adma.201703185.
